# Genome-wide oxidative bisulfite sequencing identifies sex-specific methylation differences in the human placenta

**DOI:** 10.1080/15592294.2018.1429857

**Published:** 2018-02-21

**Authors:** Sungsam Gong, Michelle D Johnson, Justyna Dopierala, Francesca Gaccioli, Ulla Sovio, Miguel Constância, Gordon CS Smith, D Stephen Charnock-Jones

**Affiliations:** a Department of Obstetrics and Gynaecology, University of Cambridge, NIHR Cambridge Comprehensive Biomedical Research Centre, Cambridge, CB2 0SW, United Kingdom; b Centre for Trophoblast Research (CTR), Department of Physiology, Development and Neuroscience, University of Cambridge, Cambridge, CB2 3EG, United Kingdom

**Keywords:** whole-genome oxidative bisulfite sequencing, placenta, DNA methylation, sex-specific methylation differences, RNA-sequencing, *CSMD1*, differentially methylated region

## Abstract

DNA methylation is an important regulator of gene function. Fetal sex is associated with the risk of several specific pregnancy complications related to placental function. However, the association between fetal sex and placental DNA methylation remains poorly understood. We carried out whole-genome oxidative bisulfite sequencing in the placentas of two healthy female and two healthy male pregnancies generating an average genome depth of coverage of 25x. Most highly ranked differentially methylated regions (DMRs) were located on the X chromosome but we identified a 225 kb sex-specific DMR in the body of the *CUB and Sushi Multiple Domains 1* (*CSMD1*) gene on chromosome 8. The sex-specific differential methylation pattern observed in this region was validated in additional placentas using in-solution target capture. In a new RNA-seq data set from 64 female and 67 male placentas, *CSMD1* mRNA was 1.8-fold higher in male than in female placentas (*P* value = 8.5 × 10^−7^, Mann-Whitney test). Exon-level quantification of *CSMD1* mRNA from these 131 placentas suggested a likely placenta-specific *CSMD1* isoform not detected in the 21 somatic tissues analyzed. We show that the gene body of an autosomal gene, *CSMD1*, is differentially methylated in a sex- and placental-specific manner, displaying sex-specific differences in placental transcript abundance.

## Introduction

The placenta is a crucial, yet short-lived, organ at the interface between mother and the developing fetus. Among its many functions, it mediates nutrient uptake and gas and waste exchange, and acts as protective barrier against some pathogens. Many adverse pregnancy outcomes, such as stillbirth, neonatal death, growth restriction, cerebral palsy (due to oxygen deprivation at birth), and preterm labor can be attributed to compromised placental function [1]. Moreover, the intrauterine environment is also a key determinant of the risk for cardiovascular disease in adult life [2]. Fetal sex is itself often associated with increased risk for specific pregnancy complications [3] and, since the placenta is a fetal organ, it becomes a suitable tissue to study the molecular mechanisms that may underlie sex-specific adverse pregnancy outcomes.

DNA methylation is one of the most intensely studied epigenetic regulatory mechanisms and determines cellular phenotypes, commonly through the regulation of transcript abundance. Previous studies have demonstrated the role for DNA methylation in placental genomic imprinting and X inactivation [4-6]. Studies that applied whole-genome bisulfite sequencing to analyze the placental methylome have either focused on understanding genomic imprinting [7] or been limited to the analysis of a single placenta [8] and could not, therefore, address sex-specific patterns. Although a few studies have examined sex-specific differences in DNA methylation, they have either assayed DNA methylation for only 1–2% of CpG sites [9] or specifically focused on X chromosome differences [4].

Given the vital role of the placenta in maintaining a healthy pregnancy and the association between fetal sex and specific pregnancy complications, we sought to characterize sex-specific differences in global DNA methylation and identify sex-specific differentially methylated regions from placental whole-genome oxidative bisulfite sequencing data sets (WGoxBS). We tested the hypothesis that sex-specific differential methylation is associated with transcript abundance using an extensive placental RNA-seq data set.

## Results

### Genome-wide methylation differences by fetal sex

**Figure 1.**
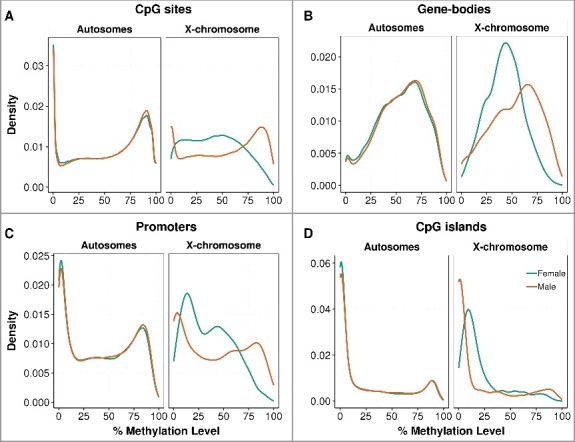
Distribution of placental methylation level by fetal sex. The percentage methylation level was measured in autosomes separately from the chromosome X at (A) single CpG sites, (B) gene-bodies, (C) promoters, and (D) CpG island regions. The distribution curves were plotted by the kernel density estimation, where the area under the curve equals to one. The distributions of genome-wide methylation levels are shown in Supplementary Figure S2 and the methylation levels are described in Supplementary Table S3.

We carried out whole-genome oxidative bisulfite sequencing (WGoxBS) in two female and two male term placentas from well-characterized healthy pregnancies of the Pregnancy Outcome Prediction Study Cohort [10-12] (Supplementary Table S1). We obtained a total of 1.7 billion uniquely mapped reads, this equates to an average of 26x genome coverage per sample (Supplementary Table S2). For our initial analyses, we used the 14,960,649 strand-specific CpG sites that were sequenced at a depth of at least 10x and shared among all four individuals (Supplementary Figure S1). As previously documented [8], the placenta has lower methylation levels than other tissues, with a global CpG methylation value of 53.5% (autosomes: 53.6%, X-chromosome: 46.6%). Methylation of single CpG sites showed a typical bimodal distribution pattern (Figure 1A), as previously reported [8,13], whereas CpG islands (Figure 1D) were globally hypomethylated, as expected [14]. Gene bodies (Figure 1B) were the most methylated regions at 54%, followed by CpG island shores (47%), enhancers (35%), promoters (20%) (Figure 1C), and CpG islands (16%). In addition to finding that male placentas were 1.6% more methylated than female placentas, lowly methylated (<50%) single CpG sites, gene bodies, and promoter regions were more common in females (Figure 1A-1D). This global male-hypermethylation was reproduced using an in-solution target capture methodology (SureSelect) for the two samples assayed by WGoxBS (technical replicates; 1% more methylated in male). It was also reproduced in six additional samples, three from female placentas and three from male placentas (i.e., biological replicates; 1.5% more methylated in male) (Supplementary Table S3). However, further investigation of eight other tissues from the Schultz et al. [15] datasets suggests this male hypermethylation could be tissue-specific, as female-hypermethylation is observed in the adrenal and spleen (Supplementary Table S3).

**Figure 2.**
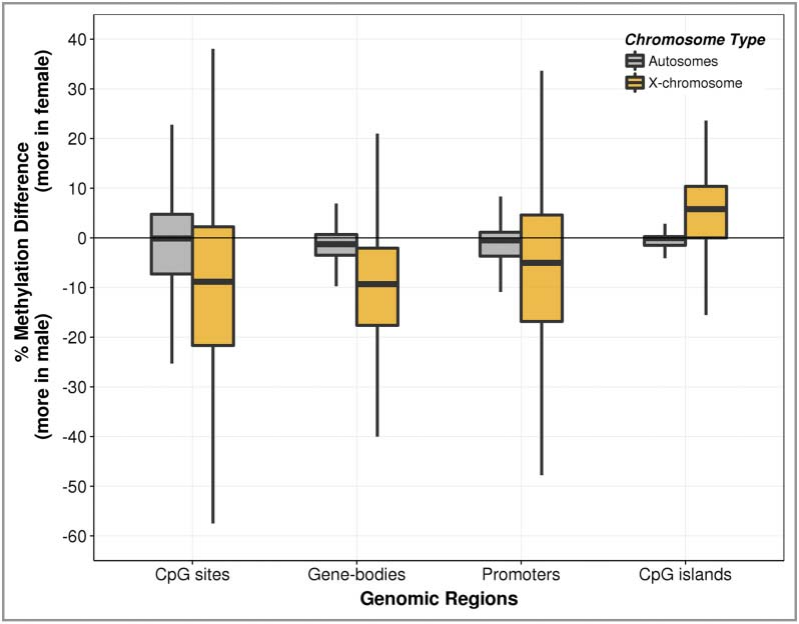
Boxplot of methylation difference by fetal sex. The CpG methylation differences were obtained from the percentage methylation level of females (n = 2) subtracted by that of males (n = 2) at single CpG sites, gene-bodies, promoters, and CpG island regions. The methylation differences were measured in autosomes separately from the chromosome X. Each of the boxes shows the median and interquartile range (IQR). The vertical lines (whiskers) extended from the box represent a range of 1.5*IQR from both ends. Data beyond the end of the whiskers (i.e., outliers) are not shown. Boxplots of chromosome-wide methylation difference are shown in Supplementary Figure S3.

Having observed global placental methylation differences between the sexes, we investigated the chromosome-wide methylation differences between females and males (Figure 2 and Supplementary Figure S3). Chromosome X CpGs were globally hypomethylated in females, whereas there was little evidence for sex-specific differences in overall methylation in the autosomes (Figure 2). Next, we determined whether this hypomethylation of the X chromosome in females varied according to the genomic feature. We found that the X chromosome was globally hypomethylated in females, except at CpG islands, where females were hypermethylated compared to males; this was also reproduced in the SureSelect datasets (Supplementary Figure S4).

### Sex-specific differentially methylated regions

**Figure 3.**
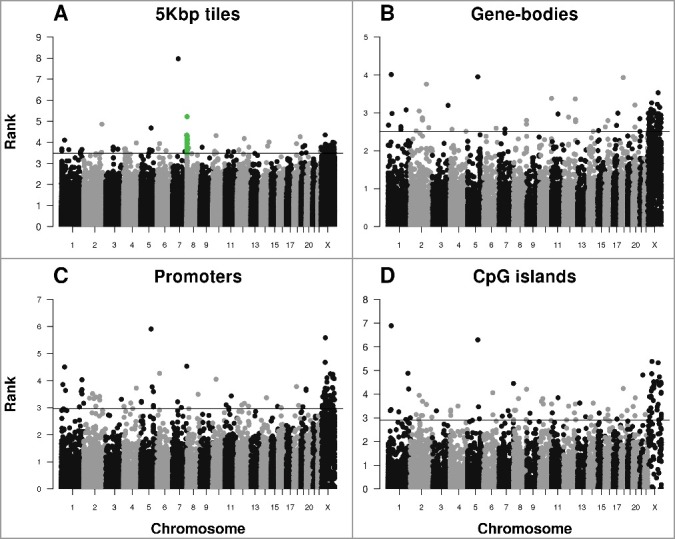
Manhattan plot of highly ranked sex-specific differentially methylated regions. Each dot represents a (A) a tile of 5 kb, (B) gene-body, (C) promoter, and (D) CpG island region. Dots above the horizontal lines represent top 100 highly ranked DMRs within each figure. The green dots in (A) represent 20 top ranked DMRs within *CSMD1*. The rank in Y-axis is converted from the combined rank of RnBeads (see Methods).

In order to identify sex-specific differentially methylated regions (DMRs), we analyzed our data using the Bioconductor package RnBeads [16]. We created Manhattan plots analyzing DMRs by 5 kb tiles, gene bodies, promoters, and CpG islands (Figure 3A-3D). Consistent with the chromosome specific analysis of global methylation, most highly ranked DMRs were located on the X chromosome (Supplementary Table S4). For autosomal regions, apart from isolated regions with highly ranked sex-specific DMRs, there was strong evidence for a cluster of multiple sex-specific DMRs located on chromosome 8 (Figure 3A).

**Figure 4.**
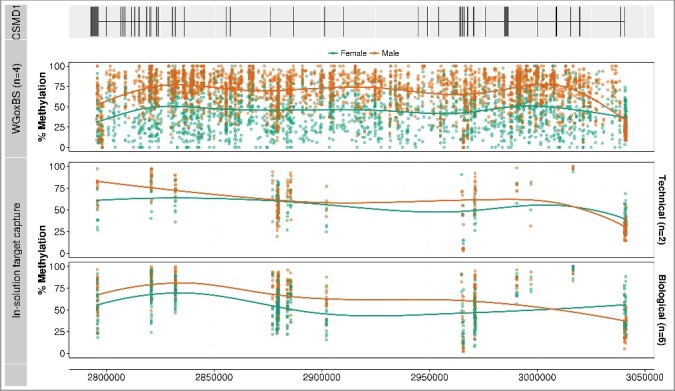
Methylation profiles of the 3’ region of the *CSMD1* locus. The figure focuses on the 3’ end of *CSMD1*, where the 20 differentially methylated 5 kb regions (8:2,795,000-3,020,000) are located. The first track shows the gene structure of *CSMD1*—the exons are represented by vertical lines and the introns by a horizontal line. The second panel displays methylation profiles from the WGoxBS samples, where each dot represents one CpG site for each individual (male in orange and female in green). The third panel shows the same samples analyzed by in-solution target capture. The fourth panel shows independent biological replicate samples analyzed using in-solution capture. Placental WGoxBS samples show hypomethylation in females (green), which is technically and biologically validated using an in-solution target capture method. The figure is drawn using ggbio Bioconductor package [17].

The most highly ranked 5 kb DMR on chromosome 8 shows average methylation values of 36% and 72% in females and males, respectively. This DMR and 19 additional 5 kb DMRs ranked amongst the top 100 differentially methylated regions (Supplementary Table S4). All 20 DMRs were located within a 225 kb locus (8:2,795,000-3,020,000) of the *CUB and Sushi Multiple Domains 1* (*CSMD1*, 8:2,792,875-4,852,494) gene, which is located on the minus strand (Figure 4). Consistent with the highest ranked chromosome 8 DMR, the average methylation value across these twenty 5 kb DMRs was 42% and 71% in females and males, respectively.

We then studied the *CSMD1* and X chromosome DMRs using SureSelect technical (n = 2) and biological replicates (n = 6) (Supplementary Table S1). The in-solution target capture methodology yielded 2.4 and 1.3 million strand-specific CpG sites that were sequenced at a depth of at least 10x and shared among the two technical and six biological replicates, respectively (Supplementary Table S5). We observed good concordance (*R*^2^ = 0.81 females, *R*^2^ = 0.95 males) in the level of methylation between technical and biological replicates of X chromosome CpGs present in the 100 top-ranked 5 kb tile, gene-body, promoter, and CpG island regions shown in Figure 3A-3D (Supplementary Table S4 and Figure S5).

Females were consistently and significantly hypomethylated compared to males over the extensive differentially methylated region of *CSMD1* (Figure 4). With the in-solution target capture methodology, the level of methylation of individual CpG sites within these 20 chromosome 8 DMRs was statistically significantly different between females and males, both for the technical and biological replicates (*P* = 9.2 × 10^−38^ and *P* = 1.1 × 10^−29^, respectively, Fisher's Exact test). More importantly, the average level of methylation within the chromosome 8 DMR in females and males (8:2,795,000-3,020,000) was consistent among all three data sets: WGoxBS (47% vs. 71%, n = 4), technical replicates (52% vs. 72%, n = 2), and biological replicates (52% vs. 68%, n = 6). Having validated this extensive sex-specific DMR, we then determined whether this sex-specific methylation pattern is placenta-specific or observed in other tissues. We obtained data from Schultz et al. [15] and studied the same region in 8 somatic tissues, comparing methylation levels in 1 male and 1 female. Interestingly, sex-specific differential methylation of the *CSMD1* region was only observed in the placenta and not in the other 8 tissues studied (Supplementary Figure S6). The average level of methylation in females and males in these tissues was 82% and 81%, respectively. We also examined the methylation profile in 6 other tissues obtained from the NIH Roadmap Epigenomics Mapping Consortium [18]. Although only a single male [brain germinal matrix (87%), hippocampus (89%), testis spermatozoa (84%)] and a single female [fetal muscle (79%), fetal thymus (87%), breast luminal epithelium (80%)] sample were available, no obvious male-female methylation differences were observed. The 14 non-placental tissues were highly methylated at this locus (range: 79% to 89%), regardless of the sex and the site where the bisulfite sequencing data was generated (Supplementary Figure S6).

### Sex-specific *CSMD1* differential methylation and transcript abundance

**Figure 5.**
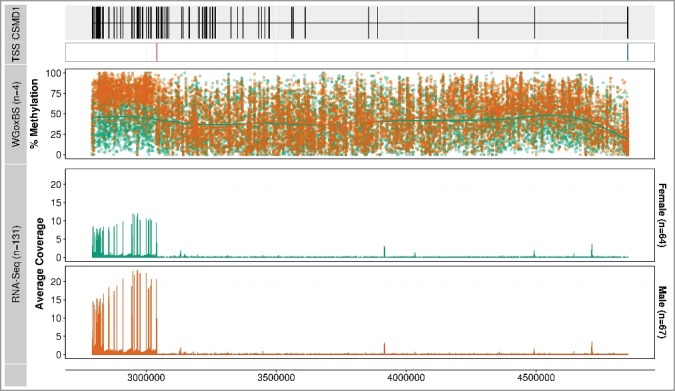
Methylation profiles and RNA-seq coverage over the *CSMD1* locus. The first track displays the gene structure of the entire *CSMD1* locus (8:2,792,875-4,852,494) and the second panel shows the methylation profile of the WGoxBS samples (n = 4), where each dot represents one CpG site for each individual. The third track displays the mean RNA-seq coverage of 64 female and 67 male placental samples. Note that most of the RNA-seq reads are aligned toward the 3’ end, where the differentially methylated regions are located (displayed in Figure 4). Canonical and placenta-specific transcription start sites are shown in blue and red, respectively.

To understand the potential functional significance of this extensive and sex-specific differentially methylated region of *CSMD1* in the placenta, we quantified the abundance of *CSMD1* RNA from a placental RNA-seq data set generated from 64 healthy female and 67 healthy male placentas (Supplementary Tables S6 and S7). At the gene level, female samples had 1.8-fold lower transcript abundance than male samples (*P* = 8.5 × 10^−7^, Mann-Whitney test). As transcript abundance typically is negatively correlated with the level of methylation in the promoter [19], we compared the methylation level of the annotated *CSMD1* promoter region (8:4,851,994-4,853,994) in female and male placental samples. We did not observe sex-specific methylation differences at the promoter region (Supplementary Figure S7). Intriguingly, we noted that most of the placental RNA-seq reads aligned towards the 3’ end of the *CSMD1* gene, the same region where the 20 differentially methylated regions were identified (Figure 5). The first canonical exon [i.e., first exon near the annotated transcription start site (TSS), 8:4,851,994)] of *CSMD1* did not have a single placental RNA-seq read. This suggests that the *CSMD1* placental transcript does not start with the canonical TSS.

Using RNA-seq data from 131 individual placentas, we reconstructed the *CSMD1* transcript. This showed that the first exon, and thus the TSS, is actually 1.8 mega base pairs downstream the canonical first exon (Supplementary Figure S8). This suggests that the placenta uses an alternative promoter that is different from the reference gene annotation (e.g., GENCODE). Then, we re-examined the methylation level at the likely promoter region (1,500 bp upstream and 500 bp downstream the putative TSS) and found that females had higher methylation values (38%) compared to males (23%) in the WGoxBS data set (*P* = 1.7 × 10^−13^, Supplementary Figure S9). This pattern of higher methylation in females was also validated technically (38% vs. 29%) and biologically (48% vs. 29%) using the in-solution target capture methodology (*P* = 1.7 × 10^−86^ and *P* = 1.5 × 10^−65^, respectively, Fisher's Exact test, Supplementary Figure S9). In addition, the putative promoter region is annotated as a DNase hypersensitive region, a transcription factor binding site, and a transcription start site (5' cap analysis gene expression, CAGE), which suggest the putative promoter region is biologically relevant (Supplementary Figure S10). This region was hypermethylated (>79%) in both sexes in all eight tissues from Schultz et al. (Supplementary Figure S11).

### *CSMD1* placenta-specific isoform

**Figure 6.**
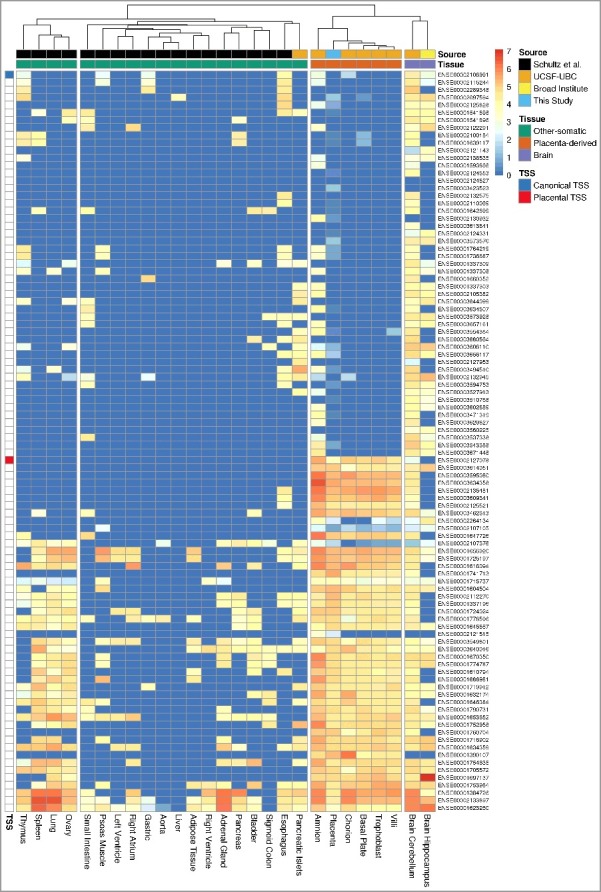
Hierarchical clustering of tissues based on the relative expression level of *CSMD1* exons. Each column represents a tissue and each row represents an Ensembl *CSMD1* exon. The relative expression is measured in the log-scale of FPKM (see Methods) and it is colored from red (high) to dark blue (low). The columns (tissues) are hierarchically clustered by the similarity of the expression pattern and the rows (exons) are ordered by the position of exons within the *CSMD1* transcript: transcript-start (top) to transcript-end (bottom). Except the placenta (this study), the RNA-Seq datasets of other tissues are from the following three participating members of NIH Roadmap Epigenomics Mapping Consortium: Schultz et al. (GEO accession: GSE16256), UCSF-UBC (GEO accession: GSE16368), and Broad Institute (GEO accession: GSE17312). Exons that contain canonical and placenta-specific transcription start site (TSS) are shown in blue and red, respectively.

We further investigated whether this potential isoform of *CSMD1* is specific to the placenta. We obtained RNA-seq data of five placenta-derived tissues from the NIH Roadmap Epigenomics Mapping Consortium (REMC) [18], 18 somatic tissues from Schultz et al. [15], and three additional tissues from the REMC, respectively (Supplementary Table S8). We quantified *CSMD1* mRNA abundance at the exon-level and compared the placenta-related tissues, including our own data, with the other 21 tissues. All the placenta-derived tissues clustered separately from the 21 non-placental tissues and they shared the same exon (ENSE00002127078), which is the first exon covered with at least 8 uniquely mapped reads (Figure 6).

This is the candidate first exon predicted as an alternative transcript start site by the transcript reconstruction using our 131 RNA-seq samples. Interestingly, none of the other non-placental tissues, except the cerebellum, have reads covering this exon, suggesting that they do not use this putative first exon. We therefore propose that the placental transcript of *CSMD1* has a different TSS from other tissues and it is a novel isoform specific to the placenta. The exon usage comparison with other fetal and embryonic tissues [20] also supports this putative TSS and a novel form of placenta *CSMD1* transcript (Supplementary Figure S12).

The putative protein encoded by the placental-specific transcript is predicted to contain 4 CUB and 18 Sushi extracellular domains instead of the full 14 CUB and 28 Sushi domains, as expected from the reference sequence (Supplementary Figure S13). *CSMD1* exon-level expression analysis from the 64 female and 67 male placental RNA-seq data identified 15 exons that were significantly differentially expressed (*P*<0.05, Mann-Whitney test) in a sex-specific manner (Supplementary Table S9). Consistent with the gene-level quantification, females showed lower expression compared to males, from 14 exons out of the 15 significantly differentially expressed exons.

## Discussion

Whole-genome oxidative bisulfite sequencing (WGoxBS) was used as a discovery method to assay CpG methylation at single nucleotide resolution in two female and two male healthy term placentas with an average of 26x genome coverage per individual yielding a data set of ∼15 million CpGs (Supplementary Table S2 and Supplementary Figure S1). The majority of methylation studies of the placenta have used Illumina BeadChip arrays, which assay up to 450,000 CpGs, or have focused on identifying differences between the placentas of healthy and complicated pregnancies (reviewed by Bianco-Miotto et al.  [9]). As a discovery method, we carried out WGoxBS with validation in technical and biological replicates using in-solution target capture bisulfite sequencing. Finally, using an RNA-seq data set generated from 64 female and 67 male healthy term placentas we determined gene- and exon-level transcript abundance associated with a 225 kb sex-specific DMR located within the *CSMD1* gene.

In agreement with other studies, we showed that placental DNA is hypomethylated compared to that from other somatic tissues [4,21,22]. After examining the global methylation profiles within various genomic features, we observed that males are generally hypermethylated compared to females, and the differences are most evident on the X chromosome. There is an exception, however, to this global male hypermethylation, namely, X chromosome CpG islands hypermethylated in females. Our own analysis of eight tissues from the whole-genome bisulfite sequencing data of Schultz et al. showed that X chromosome hypermethylation in CpG islands of females is not unique to the placenta. Female hypermethylation of X chromosome CpG island regions seems to be a common finding among different tissues, which was reported in a number of other studies of X chromosome inactivation, DNA methylation role at CpG island regions, and meta-analysis of human peripheral blood leukocytes [23-26].

Many (35) of our top 100 sex-specific 5 kb DMRs are on the X chromosome. A similar observation was previous reported by Yousefi et al. [27] The authors found, in umbilical cord blood, that the majority (74%) of gender-associated significantly differentially methylated CpGs were located on the X chromosome, with 64% of them being hypermethylated in females. Also, a recent paper reports a similar observation [28]. Surprisingly, we found that 20 of the top 100 sex-specific 5 kb DMRs were located within a 225 kb region of the gene-body of *CSMD1*, with females being hypomethylated. The differential methylation in this large region was not evident when analyzing individual CpGs, CpG islands, or promoters. This suggests that the use of 5 kb tiles alongside these other methods is able to reveal additional chromosomal features that may be of functional relevance. This also highlights the advantage of using whole-genome analysis, as the number of CpGs evaluated is higher (∼30-fold in this study) than when using current array-based methods. This observation was confirmed using technical and biological replicates. We did not observe sex-specific methylation of *CSMD1* in any of the eight somatic tissues, which suggests that this sex-specific differential methylation is restricted. Demonstration of true placental specificity will require systematic analysis of this *CSMD1* DMR in all tissues and developmental stages in replicate female and male samples.

Schultz et al. applied whole-genome bisulfite sequencing (WGBS), a method in which 5-methylcytosine and 5-hydroxymethylcytosine are indistinguishable; therefore, the two modifications are reported in aggregate [29,30]. As WGoxBS measures 5-methylcytosine alone [31,32], we were able to detect the *CSMD1* DMR in the absence of 5-hydroxymethylcytosine. Despite our discovery data set being derived from WGoxBS, we technically validated our sex-specific *CSMD1* DMR with an in-solution methodology using bisulfite conversion alone. We also validated this observation in 3 additional healthy female and male placentas (biological replicates) using the same technique. Thus, we are confident that the placental and sex-specific *CSMD1* DMR is due to differences in 5-methylcytosine and not 5-hydroxymethylcytosine.

We analyzed *CSMD1* RNA-seq reads obtained from 64 female and 67 male placental samples and showed a sex-specific difference in transcript abundance, where females have lower transcript abundance compared to males. Our analysis of transcript reconstruction based on the 131 placental RNA-seq samples suggests that the placenta may have a novel *CSMD1* transcript that starts 1.8 mega base pairs downstream the canonical first exon. While no sex-specific methylation differences were detected in the canonical promoter, females had higher methylation values in the newly identified putative promoter region, which may explain overall decreased transcript abundance in females when compared to males. This putative placental promoter region was hypermethylated (>85%) in the eight tissues from the Schulz et al. dataset with no evidence of use of the putative first placental exon. While sex-specific transcript abundance of *CSMD1* can be explained by differences in DNA methylation at the putative promoter region, the 225 kb *CSMD1* DMR, located 20 kb downstream the putative placental-specific first exon, might also affect gene-level transcript abundance.

Comparative analysis of RNA-seq data of *CSMD1* at the exon-level between the placenta-derived tissues and other adult and fetal tissues indicates that the placenta has a different first exon and its location is consistent with our proposed model of a novel transcript. The Ensembl genome browser annotates that one of the *CSMD1* transcripts (ENST00000520451) starts with the same exon (ENSE00002127078), which we identified as the putative first exon in the placenta-derived tissues. However, the Ensembl transcript consists of only five exons with no evidence of an open reading frame, whereas our model of a novel placental *CSMD1* transcript spans 35 exons and its protein-coding potential is highly marked by the Coding-Potential Assessment Tool [33]. However, very little is known about the possible function of CSMD1—it has neither a Gene Ontology biological process nor a molecular function term associated with it and the cellular components terms (“membrane” and “integral component of membrane”) are only inferred from electronic annotation. Nonetheless, it has been linked in genome-wide association studies with Parkinson's disease, Alzheimer's disease, and schizophrenia (for an example see [34]). A fragment of recombinant rat *CSMD1* inhibited the classical but not the alternative complement activation pathways [35]. A recent study showed that suppression of *CSMD1* by short hairpin RNAs significantly increased the proliferation, cell migration, and invasiveness of MCF10A cells compared to controls [36]. It is not known what possible role *CSMD1* might have in the placenta, but regulation of complement [37] and cell invasiveness are known to be important [38].

The 225 kb DMR described above covers the exons (and introns) that comprise this novel transcript, which is also differentially expressed in male and female placentas. Gene body methylation is an informative predictor of gene activity [39], although detailed mechanistic understanding of this, and particularly how it may differ between males and females, is currently lacking.

Whole-genome oxidative bisulfite sequencing and in-solution target capture carried out in the placentas of healthy female and male replicates allowed us to identify and validate a sex-specific autosomal 225 kb differentially methylated region in the *CSMD1* gene which was hypermethylated in the male placenta. RNA-seq analysis of 64 healthy female and 67 healthy male placenta samples showed that *CSMD1* had sex-specific differential transcript abundance (∼1.8-fold higher in male). Analysis of DNA methylation and RNA-seq data from publically available data sets indicate that the differential methylation is likely to be placental-specific, with the presence of a placental-specific *CSMD1* isoform. Further studies will be required to elucidate the cause, consequences, and role for sex-specific differential methylation, transcript abundance, and placental-specific isoforms of *CSMD1* in the placenta. These data illustrate the benefit of analyzing comprehensive RNA-seq data in parallel with DNA methylation data.

## Material and methods

### Placental samples

All samples were obtained as part of The Pregnancy Outcome Prediction study (POPs)—a prospective cohort study of nulliparous women attending the Rosie Hospital, Cambridge (UK). The study and sample collection have been previously described in detail [10-12]. Ethical approval for the study was obtained by the Cambridgeshire 2 Research Ethics Committee (reference number 07/H0308/163). All participants gave written informed consent.

### DNA extraction

Frozen placental tissue samples from four separate regions from the basal plate of each term placenta were combined to provide a single 20-24 mg sample for DNA extraction. This was processed using the Qiagen DNeasy Blood & Tissue Kit (Qiagen), according to the manufacturer's instructions including the RNase treatment. To achieve buffer compatibility for oxidative whole-genome bisulfite conversion, placental DNA was eluted in UltraPure water (Cambridge Epigenetix). All DNA samples were quantified using the Qubit® dsDNA HS Assay Kit (Life Technologies).

### Whole-genome oxidative bisulfite sequencing

Placental genomic DNA (4 µg) from 4 healthy pregnancies was processed to achieve 10 kb fragments with the g-Tube (Covaris), according to the manufacturer's instructions. Fragmented DNA was concentrated using GeneJET purification columns, according to the manufacturer's instructions (Thermo Fisher Scientific). This fragmented and purified DNA (1.5 µg) was taken forward for oxidation + bisulfite treatment using the TrueMethyl Kit (Cambridge Epigenetix). The supplied sequencing and digestion controls were added at least to 1% (w/w) to the fragmented DNA. Buffer exchange, denaturation, oxidation, bisulfite conversion, cleanup, and qualitative assessment of 5-hydroxymethylcytosine oxidation was carried out according to manufacturer's instructions (version 3.1). Bisulfite-converted DNA (50 ng) was used as input for library generation using the EpiGnome™ Methyl-Seq Kit (Epicentre) and EpiGnome™ Index PCR Primers (Epicentre), according to manufacturer's instructions. All double and single stranded DNA was quantified using the Qubit dsDNA HS Assay Kit and Qubit ssDNA Assay Kits, respectively, according to the manufacturer's instructions (Life Technologies), and were sized with the Agilent Bioanalyzer 2100 High Sensitivity DNA assay. Molarity of each of the libraries was determined by qPCR using the KAPA Illumina ABI Prism Library Quantification Kit (Kapa Biosystems). Indexed libraries were pooled at an equimolar ratio. To increase the number of uniquely sequenced reads, two independent libraries were generated for each individual. Multiplexed sequencing was carried out on the Illumina MiSeq, HiSeq 2000, and HiSeq 2500 instruments with 2 × 100, 2 × 150 and 2 × 125 cycles using MiSeq Reagent Kit v3, HiSeq SBS Kit v3 and HiSeq SBS Kit v4, respectively. The methylation conversion rate of the supplied sequencing controls was checked with bsExpress (https://bitbucket.org/cegx-bfx/cegx_bsexpress_docker).

### In-solution target capture library preparation and sequencing

Placental genomic DNA (3.5 µg) from 8 healthy pregnancies (including 2 from the WGoxBS) was fragmented by the Covaris S220 system according to the SureSelect Methyl-Seq target enrichment protocol (Agilent). As oxidative bisulfite sequencing was unsupported, library preparation, hybridization, bisulfite conversion, indexing, and sample pooling were carried out according to manufacturer's instructions. After the 3’ end adenylation step, we excluded DNA fragments >500 bases by performing an AMPure XP bead selection with a 0.6x bead to DNA ratio discarding the larger fragments that were bound by the beads. We continued the prescribed purification by adding an additional 1.2x volume beads to the supernatant to bring the final ratio to 1.8x. Resultant libraries were sized with the Agilent Bioanalyzer 2100 High Sensitivity DNA assay and molarity of each library was determined by qPCR using the KAPA Illumina ABI Prism Library Quantification Kit (Kapa Biosystems). All 8 libraries were pooled and sequenced on the Illumina HiSeq 2500 instrument with 2 × 125 cycles using HiSeq SBS Kit v4 and a single lane of the Illumina HiSeq 4000 instrument with 2 × 150 cycles using HiSeq 3000/4000 SBS Kit following Illumina's guidelines (Illumina Application Note: Epigenetics February 2016).

### Placental biopsies and RNA extraction

Placental biopsies (n = 64 female placentas, n = 67 male placentas) were selected from healthy pregnancies from the POPs cohort. These patients had no evidence of hypertension at booking and during pregnancy, did not experience pre-eclampsia, Hemolysis, Elevated Liver enzymes, and Low Platelets (HELLP) syndrome, gestational diabetes, or diabetes mellitus type I or type II and other obstetric complications. They delivered live babies with a birth weight percentile in the normal range (20-80^th^ percentile), with no evidence of slowing in fetal growth trajectory. Chorionic villi from the corresponding placentas (free from decidua, visible infarction, calcification, hematoma, or damage) were collected and processed within 30 minutes of separation from the uterus. After repeated washes in chilled phosphate buffered saline, the samples were placed in RNA later (Applied Biosystems) and stored at -80°C. Total placental RNA was extracted using mirVana Isolation Kit (Ambion). For each placenta, approximately 5 mg of tissue were homogenized in the Lysis/Binding solution for 20 sec at 6 m/s using a bead beater (FastPrep24) and Lysing Matrix D Tubes (MP Biomedicals). The samples were then spun at 13,000 rpm for 5 min at 4°C and the supernatants recovered. Afterwards, the manufacturer's instructions were followed. Immediately after the RNA extraction, placental RNA samples were DNase-treated using DNA-free DNA Removal Kit (Ambion), aliquoted, and stored in -80°C. Quantity and quality of the RNA samples were assessed using the Agilent 2100 Bioanalyzer, the Agilent RNA 6000 Nano Kit (Agilent Technologies), and Qubit fluorometer.

### Library preparation for RNA-Seq

Libraries were prepared starting with 300-500 ng of good quality total RNA (RIN ≥7.5) using the TruSeq Stranded Total RNA Library Prep Kit with Ribo-Zero Human/Mouse/Rat (Illumina), according to the manufacturer's instructions. The kit contains 96 uniquely indexed adapter combinations in order to allow pooling of multiple samples prior to sequencing. After determining their size (with the Agilent 2100 Bioanalyzer and the Agilent High Sensitivity DNA Kit by Agilent Technologies) and concentration (by qPCR with the KAPA Illumina ABI Prism Library Quantification Kit, Kapa Biosystems), libraries have been pooled and sequenced (single-end, 125 bp) using a Single End V4 Cluster Kit and an Illumina HiSeq2500 or HiSeq4000 instrument.

### Sequencing data processing and analysis

Both WGoxBS and in-solution target capture data went through the quality control process (i.e., trimming primer sequences and poor quality bases toward 3’ end) by Trim Galore! [40] after checking the quality metrics of raw reads with FastQC [41]. Then, the high quality trimmed reads were mapped to the human genome reference (hg19) using Bismark [42]. Reads of poor mapping quality (MAPQ = 0) and duplicated reads (i.e., those mapped at the same start and end position within the same chromosome) were removed from the alignment files before calling methylated CpGs by Bis-SNP [43], which is a genotype-aware methylcytosine caller enabled with a base-quality recalibration function built on top of the Genome Analysis Toolkit [44]. The VCF files generated by Bis-SNP were converted into BED format as inputs for RnBeads [16] to find differentially methylated regions. RnBeads considers both the statistical significance (i.e., *P* value) and the effect size (i.e., absolute and relative methylation difference) and takes the worst rank (combined rank) from the three individual ranks of aforementioned measurements. In Manhattan plots (Figure 3), to make highly ranked DMRs (smaller values) appear in top, the original combined rank is converted by subtracting the log-scale of combined rank from the log-scale of maximum rank. CpGs of at least 10x coverage and shared among all four individuals were considered and DMRs were filtered if they had fewer CpG sites compared to the median of the CpGs found in the dataset: 17 (5 kb tiles), 61 (gene-bodies), 18 (promoters), and 51 (CpG islands).

The definitions of CpG island regions and the gene model were from the UCSC (hg19) table browser [45] and the Ensembl 73 equivalent with the GENCODE version 18, respectively, which were accompanied with RnBeads package. The promoter regions were defined as 1,500 bp upstream and 500 bp downstream regions of the transcription start sites. The CpG island shore regions were defined as 2,000 bp flanking regions from both ends of the CpG island regions. The ‘5,000bp’ regions (5 kb) are defined as a set of genomic regions where each chromosome is tiled by non-overlapping 5 kb windows.

Technical and biological validation of the sex-specific methylation patterns was performed using Fisher's exact test on a 2 × 2 contingency table by testing independence of sex and the cumulative count of methylated CpGs over the 5,000 bp region. For technical validation, one female and one male sample were tested by both WGoxBS and in-solution capture methodologies. For biological validation, all 3 female and all 3 male samples assayed by the in-solution capture methodology were used. A two-sided P value <0.05 was considered statistically significant.

The Schultz et al. [15] methylation data for 8 tissues [adrenal gland (AD), aorta (AO), esophagus (EG), adipose tissue (FT), gastric (GA), psoas muscle (PO), small bowel (SB), and spleen (SX)] were downloaded from http://neomorph.salk.edu/SDEC_tissue_methylomes/processed_data/allc_tissues.tar. CpGs with at least 10x coverage detected in individual 2 (a female) and individual 3 (a male) were used. The cytosine methylation level was measured as recommended by Schultz et al. [46]. Briefly, for single CpG sites, the ratio of reads with methylation (C base) out of the total number of reads covering the position (sum of C and T reads) was calculated; within regional contexts (e.g., gene-body, CpG island, promoter, etc.), the weighted methylation level (i.e., the sum of reads with methylation out of the sum of the total number reads over the region of interest) was determined. Other Roadmap [18] whole-genome bisulfite sequencing datasets generated at the Broad Institute and UCSF-UBC were downloaded from the GEO website (https://www.ncbi.nlm.nih.gov/geo/roadmap/epigenomics), as follows: fetal muscle (fMU, GSM1172596); fetal thymus (fMU, GSM1172595); hippocampus (HC, GSM1112838 and GSM916050); brain germinal matrix (GM, GSM941747); breast luminal epithelial (LE, GSM1127125), and testis spermatozoa (SP, GSM1127117). The list of tissues and downloaded file names are available in Supplementary Table S8.

A quality control process was also applied for the RNA-Seq datasets: reads were trimmed with Trim Galore!, which uses cutadapt internally [47] and were mapped to the same version of human genome reference (hg19). TopHat2 [48], a splice-aware mapper built on top of Bowtie2 [49] short-read aligner, was used in the mapping process in which so-called two-pass (or two-scan) alignment protocol was applied to rescue unmapped reads from the initial mapping step [50]. In the second mapping, previously unmapped reads were re-aligned to the exon-intron junctions detected in the first-mapping by TopHat2 and were combined across all 131 placenta samples. The initial and second mapped reads were merged by samtools [51] and the uniquely mapped reads were counted by the htseq-count of HTSeq [52] against the same gene-model (Ensembl 73) used to calculate the degree of methylation at the gene-body or promoter region. The transcript abundance of *CSMD1* was measured in fragments per kilobase of transcript per million (FPKM) mapped reads using DESeq2 Bioconductor package [53]. The average depth of RNA-seq coverage was measured by taking the mean of each coverage calculated by the ‘genomecov’ of Bedtools [54]. The exon-level expression of *CSMD1* was measured by counting mapped reads against 98 unique exons (i.e., Ensembl exons with a distinct start and end position) of *CSMD1* using featureCount of subRead [50] and ‘coverageBed’ of Bedtools. The exon-level FPKM values were transformed by the following equation: log_e_(FPKM/1000+1) and used to generate the heatmap (Figure 6). The *CSMD1* transcripts were reconstructed based on the RNA-seq data obtained from the 131 placenta samples using StringTie [55] and Cufflinks [56]. The RNA-seq data from Schultz et al. [15] and two other participating institutes from the NIH Roadmap consortium (UCSF-UCB and The Broad Institute) were downloaded from the GEO with the following accession numbers: GSE16256 (Schultz et al.), GSE16368 (UCSF-UCB), and GSE17312 (Broad Institute). The list of tissues and downloaded file names are provided in Supplementary Table S8.

The summary statistics (read counts, coverage, mapping efficiencies, etc.) of the data processing for the WGoxBS, in-solution target capture methyl-Seq, and RNA-Seq data are available in Supplementary Table S2, Supplementary Table S5, and Supplementary Table S7, respectively. A list of software, version information, and non-default options used in this study is available in Supplementary Table S10. All the computational analyses were conducted using the Linux clusters at the University of Cambridge High Performance Computing Service and the Linux workstations of School of Biological Science computing service.

## Supplementary Material

Sup-mat-Genome-wide_oxidative_bisulfite_sequencing_identifies-Gong.rar

## Availability of data

The datasets supporting the conclusions of this article is available in the EGA repository https://www.ebi.ac.uk/ega/ [57] under the following accession numbers: methylation data: EGAD00001003136; RNA-seq data: EGAD00001003457. The R source code used to produce the figures of this paper is available from: https://github.com/sung/CSMD1.paper. To visualize data in the UCSC genome browser, a trackhub is available from: https://www.obgyn.cam.ac.uk/TrackHub/CamObsGynaeHub.txt

## References

[1] Brosens I, Pijnenborg R, Vercruysse L, et al. The "Great Obstetrical Syndromes" are associated with disorders of deep placentation. Am J Obstet Gynecol. 2011;204:193-201. 10.1016/j.ajog.2010.08.009. PMID:21094932 21094932 PMC3369813

[2] Barker DJ The fetal and infant origins of adult disease. Bmj. 1990;301:1111. 10.1136/bmj.301.6761.1111. PMID:2252919 2252919 PMC1664286

[3] Verburg PE, Tucker G, Scheil W, et al. Sexual dimorphism in adverse pregnancy outcomes – a retrospective australian population study 1981-2011. PloS one. 2016;11:e0158807. 10.1371/journal.pone.0158807. PMID:27398996 27398996 PMC4939964

[4] Cotton AM, Avila L, Penaherrera MS, et al. Inactive X chromosome-specific reduction in placental DNA methylation. Human Molecular Genetics. 2009;18:3544-52. 10.1093/hmg/ddp299. PMID:19586922 19586922 PMC2742397

[5] Fowden AL, Sibley C, Reik W, et al. Imprinted genes, placental development and fetal growth. Hormone Research. 2006;65(Suppl 3):50-8. 10.1159/000091506. PMID:16612114 16612114

[6] Randhawa R, Cohen P The role of the insulin-like growth factor system in prenatal growth. Molecular Genetics and Metabolism. 2005;86:84-90. 10.1016/j.ymgme.2005.07.028. PMID:16165387 16165387

[7] Hamada H, Okae H, Toh H, et al. Allele-specific methylome and transcriptome analysis reveals widespread imprinting in the human placenta. American Journal of Human Genetics. 2016;99:1045-58. 10.1016/j.ajhg.2016.08.021. PMID:27843122 27843122 PMC5097938

[8] Schroeder DI, Blair JD, Lott P, et al. The human placenta methylome. Proceedings of the National Academy of Sciences of the United States of America. 2013;110:6037-42. 10.1073/pnas.1215145110. PMID:23530188 23530188 PMC3625261

[9] Bianco-Miotto T, Mayne BT, Buckberry S, et al. Recent progress towards understanding the role of DNA methylation in human placental development. Reproduction. 2016;152:R23-30. 10.1530/REP-16-0014. PMID:27026712 27026712 PMC5064761

[10] Gaccioli F, Lager S, Sovio U, et al. The pregnancy outcome prediction (POP) study: investigating the relationship between serial prenatal ultrasonography, biomarkers, placental phenotype and adverse pregnancy outcomes. Placenta 2016;59(Supplement 1):S17–S25. 10.1016/j.placenta.2016.10.011.

[11] Pasupathy D, Dacey A, Cook E, et al. Study protocol. A prospective cohort study of unselected primiparous women: the pregnancy outcome prediction study. BMC Pregnancy and Childbirth. 2008;8:51. 10.1186/1471-2393-8-51. PMID:19019223 19019223 PMC2611961

[12] Sovio U, White IR, Dacey A, et al. Screening for fetal growth restriction with universal third trimester ultrasonography in nulliparous women in the Pregnancy Outcome Prediction (POP) study: a prospective cohort study. Lancet. 2015;386:2089-97. 10.1016/S0140-6736(15)00131-2. PMID:26360240 26360240 PMC4655320

[13] Harris RA, Wang T, Coarfa C, et al. Comparison of sequencing-based methods to profile DNA methylation and identification of monoallelic epigenetic modifications. Nat Biotechnol. 2010;28:1097-105. 10.1038/nbt.1682. PMID:20852635 20852635 PMC2955169

[14] Bird A, Taggart M, Frommer M, et al. A fraction of the mouse genome that is derived from islands of nonmethylated, CpG-rich DNA. Cell. 1985;40:91-9. 10.1016/0092-8674(85)90312-5. PMID:2981636 2981636

[15] Schultz MD, He Y, Whitaker JW, et al. Human body epigenome maps reveal noncanonical DNA methylation variation. Nature. 2015;523:212-6. 10.1038/nature14465. PMID:26030523 26030523 PMC4499021

[16] Assenov Y, Muller F, Lutsik P, et al. Comprehensive analysis of DNA methylation data with RnBeads. Nature Methods. 2014;11:1138-40. 10.1038/nmeth.3115. PMID:25262207 25262207 PMC4216143

[17] Yin T, Cook D, Lawrence M ggbio: an R package for extending the grammar of graphics for genomic data. Genome Biol. 2012;13:R77. 10.1186/gb-2012-13-8-r77. PMID:22937822 22937822 PMC4053745

[18] Roadmap Epigenomics C, Kundaje A, Meuleman W, et al. Integrative analysis of 111 reference human epigenomes. Nature. 2015;518:317-30. 10.1038/nature14248. PMID:25693563 25693563 PMC4530010

[19] Kass SU, Landsberger N, Wolffe AP DNA methylation directs a time-dependent repression of transcription initiation. Current Biology: CB. 1997;7:157-65. 10.1016/S0960-9822(97)70086-1. PMID:9395433 9395433

[20] Gerrard DT, Berry AA, Jennings RE, et al. An integrative transcriptomic atlas of organogenesis in human embryos. eLife. 2016;5 10.7554/elife.15657 PMC4996651 27557446

[21] Ehrlich M, Gama-Sosa MA, Huang LH, et al. Amount and distribution of 5-methylcytosine in human DNA from different types of tissues of cells. Nucleic Acids Research. 1982;10:2709-21. 10.1093/nar/10.8.2709. PMID:7079182 7079182 PMC320645

[22] Fuke C, Shimabukuro M, Petronis A, et al. Age related changes in 5-methylcytosine content in human peripheral leukocytes and placentas: an HPLC-based study. Annals of Human Genetics. 2004;68:196-204. 10.1046/j.1529-8817.2004.00081.x. PMID:15180700 15180700

[23] Cotton AM, Price EM, Jones MJ, et al. Landscape of DNA methylation on the X chromosome reflects CpG density, functional chromatin state and X-chromosome inactivation. Human Molecular Genetics. 2015;24:1528-39. 10.1093/hmg/ddu564. PMID:25381334 25381334 PMC4381753

[24] Migeon BR X-chromosome inactivation: molecular mechanisms and genetic consequences. Trends in Genetics : TIG. 1994;10:230-5. 10.1016/0168-9525(94)90169-4. PMID:8091502 8091502

[25] Sharp AJ, Stathaki E, Migliavacca E, et al. DNA methylation profiles of human active and inactive X chromosomes. Genome Research. 2011;21:1592-600. 10.1101/gr.112680.110. PMID:21862626 21862626 PMC3202277

[26] Weber M, Davies JJ, Wittig D, et al. Chromosome-wide and promoter-specific analyses identify sites of differential DNA methylation in normal and transformed human cells. Nat Genet. 2005;37:853-62. 10.1038/ng1598. PMID:16007088 16007088

[27] Yousefi P, Huen K, Dave V, et al. Sex differences in DNA methylation assessed by 450 K BeadChip in newborns. BMC Genomics. 2015;16:911. 10.1186/s12864-015-2034-y. PMID:26553366 26553366 PMC4640166

[28] Martin E, Smeester L, Bommarito PA, et al. Sexual epigenetic dimorphism in the human placenta: implications for susceptibility during the prenatal period. Epigenomics. 2017;9:267-78. 10.2217/epi-2016-0132. PMID:28234023 28234023 PMC5331919

[29] Huang Y, Pastor WA, Shen Y, et al. The behaviour of 5-hydroxymethylcytosine in bisulfite sequencing. PloS one. 2010;5:e8888. 10.1371/journal.pone.0008888. PMID:20126651 20126651 PMC2811190

[30] Nestor C, Ruzov A, Meehan R, et al. Enzymatic approaches and bisulfite sequencing cannot distinguish between 5-methylcytosine and 5-hydroxymethylcytosine in DNA. BioTechniques. 2010;48:317-9. 10.2144/000113403. PMID:20569209 20569209

[31] Booth MJ, Branco MR, Ficz G, et al. Quantitative sequencing of 5-methylcytosine and 5-hydroxymethylcytosine at single-base resolution. Science. 2012;336:934-7. 10.1126/science.1220671. PMID:22539555 22539555

[32] Booth MJ, Ost TW, Beraldi D, et al. Oxidative bisulfite sequencing of 5-methylcytosine and 5-hydroxymethylcytosine. Nature Protocols. 2013;8:1841-51. 10.1038/nprot.2013.115. PMID:24008380 24008380 PMC3919000

[33] Wang L, Park HJ, Dasari S, et al. CPAT: Coding-Potential Assessment Tool using an alignment-free logistic regression model. Nucleic Acids Research. 2013;41:e74. 10.1093/nar/gkt006. PMID:23335781 23335781 PMC3616698

[34] Liu W, Liu F, Xu X, et al. Replicated association between the European GWAS locus rs10503253 at CSMD1 and schizophrenia in Asian population. Neurosci Lett. 2017;647:122-8. 10.1016/j.neulet.2017.03.039. PMID:28344127 28344127

[35] Kraus DM, Elliott GS, Chute H, et al. CSMD1 is a novel multiple domain complement-regulatory protein highly expressed in the central nervous system and epithelial tissues. J Immunol. 2006;176:4419-30. 10.4049/jimmunol.176.7.4419. PMID:16547280 16547280

[36] Kamal M, Holliday DL, Morrison EE, et al. Loss of CSMD1 expression disrupts mammary duct formation while enhancing proliferation, migration and invasion. Oncol Rep. 2017;38:283-92. 10.3892/or.2017.5656. PMID:28534981 28534981

[37] Regal JF, Gilbert JS, Burwick RM The complement system and adverse pregnancy outcomes. Mol Immunol. 2015;67:56-70. 10.1016/j.molimm.2015.02.030. PMID:25802092 25802092 PMC4447554

[38] Pijnenborg R, Robertson WB, Brosens I, et al. Review article: trophoblast invasion and the establishment of haemochorial placentation in man and laboratory animals. Placenta. 1981;2:71-91. 10.1016/S0143-4004(81)80042-2. PMID:7010344 7010344

[39] Lou S, Lee HM, Qin H, et al. Whole-genome bisulfite sequencing of multiple individuals reveals complementary roles of promoter and gene body methylation in transcriptional regulation. Genome Biol. 2014;15:408. 10.1186/s13059-014-0408-0. PMID:25074712 25074712 PMC4189148

[40] Krueger F Trim Galore!: a wrapper tool around Cutadapt and FastQC to consistently apply quality and adapter trimming to FastQ files. 2012 Available online at: http://www.bioinformatics.babraham.ac.uk/projects/trim_galore/.

[41] Simon A FastQC: a quality control tool for high throughput sequence data. 2010 Available online at: http://www.bioinformatics.babraham.ac.uk/projects/fastqc.

[42] Krueger F, Andrews SR Bismark: a flexible aligner and methylation caller for Bisulfite-Seq applications. Bioinformatics. 2011;27:1571-2. 10.1093/bioinformatics/btr167. PMID:21493656 21493656 PMC3102221

[43] Liu Y, Siegmund KD, Laird PW, et al. Bis-SNP: combined DNA methylation and SNP calling for Bisulfite-seq data. Genome Biol. 2012;13:R61. 10.1186/gb-2012-13-7-r61. PMID:22784381 22784381 PMC3491382

[44] McKenna A, Hanna M, Banks E, et al. The Genome Analysis Toolkit: a MapReduce framework for analyzing next-generation DNA sequencing data. Genome Res. 2010;20:1297-303. 10.1101/gr.107524.110. PMID:20644199 20644199 PMC2928508

[45] Karolchik D, Hinrichs AS, Furey TS, et al. The UCSC Table Browser data retrieval tool. Nucleic Acids Research. 2004;32:D493-6. 10.1093/nar/gkh103. PMID:14681465 14681465 PMC308837

[46] Schultz MD, Schmitz RJ, Ecker JR 'Leveling' the playing field for analyses of single-base resolution DNA methylomes. Trends in genetics: TIG. 2012;28:583-5. 10.1016/j.tig.2012.10.012. PMID:23131467 23131467 PMC3523709

[47] Martin JA, Wang Z Next-generation transcriptome assembly. Nature Reviews Genetics. 2011;12:671-82. 10.1038/nrg3068. PMID:21897427 21897427

[48] Kim D, Pertea G, Trapnell C, et al. TopHat2: accurate alignment of transcriptomes in the presence of insertions, deletions and gene fusions. Genome Biol. 2013;14:R36. 10.1186/gb-2013-14-4-r36. PMID:23618408 23618408 PMC4053844

[49] Langmead B, Salzberg SL Fast gapped-read alignment with Bowtie 2. Nature Methods. 2012;9:357-9. 10.1038/nmeth.1923. PMID:22388286 22388286 PMC3322381

[50] Liao Y, Smyth GK, Shi W The Subread aligner: fast, accurate and scalable read mapping by seed-and-vote. Nucleic Acids Research. 2013;41:e108. 10.1093/nar/gkt214. PMID:23558742 23558742 PMC3664803

[51] Li H, Handsaker B, Wysoker A, et al. The Sequence Alignment/Map format and SAMtools. Bioinformatics. 2009;25:2078-9. 10.1093/bioinformatics/btp352. PMID:19505943 19505943 PMC2723002

[52] Anders S, Pyl PT, Huber W HTSeq–a Python framework to work with high-throughput sequencing data. Bioinformatics. 2015;31:166-9. 10.1093/bioinformatics/btu638. PMID:25260700 25260700 PMC4287950

[53] Love MI, Huber W, Anders S Moderated estimation of fold change and dispersion for RNA-seq data with DESeq2. Genome Biol. 2014;15:550. 10.1186/s13059-014-0550-8. PMID:25516281 25516281 PMC4302049

[54] Quinlan AR, Hall IM BEDTools: a flexible suite of utilities for comparing genomic features. Bioinformatics. 2010;26:841-2. 10.1093/bioinformatics/btq033. PMID:20110278 20110278 PMC2832824

[55] Pertea M, Pertea GM, Antonescu CM, et al. StringTie enables improved reconstruction of a transcriptome from RNA-seq reads. Nat Biotechnol. 2015;33:290-5. 10.1038/nbt.3122. PMID:25690850 25690850 PMC4643835

[56] Roberts A, Pimentel H, Trapnell C, et al. Identification of novel transcripts in annotated genomes using RNA-Seq. Bioinformatics. 2011;27:2325-9. 10.1093/bioinformatics/btr355. PMID:21697122 21697122

[57] Lappalainen I, Almeida-King J, Kumanduri V, et al. The European Genome-phenome Archive of human data consented for biomedical research. Nat Genet. 2015;47:692-5. 10.1038/ng.3312. PMID:26111507 26111507 PMC5426533

